# Cardiovascular Manifestations of Pseudoxanthoma Elasticum

**DOI:** 10.7759/cureus.87113

**Published:** 2025-07-01

**Authors:** Aimee Willett, Vivek V Jasti, Sean M Muir, Jay Anderson

**Affiliations:** 1 Internal Medicine, OhioHealth Riverside Methodist Hospital, Columbus, USA; 2 Medical Education, OhioHealth Riverside Methodist Hospital, Columbus, USA

**Keywords:** angioid streaks, cardiovascular disease, median arcuate ligament syndrome, multidisciplinary care, peripheral arterial diseases, pseudoxanthoma elasticum, vascular calcification

## Abstract

Pseudoxanthoma elasticum (PXE) is a rare systemic disorder of elastic fibers. The most common manifestations include characteristic papules and ocular angioid streaks. Cardiovascular complications include peripheral arterial disease, hypertension, premature coronary artery disease, and cerebrovascular disease. These associations are secondary to dystrophic elastic fibers, resulting in the calcification of small- to medium-sized arteries.

We present the case of a 22-year-old otherwise healthy female patient diagnosed with PXE following a biopsy of a neck lesion. Ophthalmologic evaluation revealed characteristic angioid streaks. The patient also reported early satiety and leg cramping, which prompted referral to gastroenterology and cardiology. Further investigation raised concern for median arcuate ligament syndrome (MALS), though the association with her PXE diagnosis remains unclear.

This case highlights the importance of systematic evaluation, screening, and multidisciplinary management of cardiovascular manifestations in PXE.

## Introduction

Pseudoxanthoma elasticum (PXE) is a rare hereditary disorder, estimated to affect one in 25,000 to 100,000 individuals, characterized by ectopic mineralization and fragmentation of elastic fibers due to mutations in the ABCC6 gene, inherited in an autosomal recessive manner [1]. It typically presents in the second or third decade of life, although clinical expression can be highly variable. Initial skin findings include yellow cobblestone papules, often on the lateral neck or axilla [2]. Ocular complications are the most frequently documented extra-dermatologic features. A “peau d’orange” appearance of the fundus may occur in childhood and is often asymptomatic. Angioid streaks develop later in life due to mineralization of the elastic layer of Bruch’s membrane between the retinal pigment epithelium and the choroid, potentially resulting in central vision distortion [3].

Cardiovascular manifestations arise from dystrophic mineralization and degeneration of elastic fibers within the internal elastic lamina and tunica media, leading to arterial stiffness and progressive calcification. Associated manifestations include narrowing of small- to medium-sized arteries, contributing to peripheral vascular disease, hypertension, cerebral microvascular disease, and premature coronary artery disease. PXE can also affect the gastrointestinal vasculature, and though rare, cases of chronic mesenteric ischemia have been reported, likely secondary to similar elastin-related vascular changes [4].

Management of PXE is tailored to its systemic manifestations. Early recognition is essential to facilitate appropriate multidisciplinary evaluation, particularly for cardiovascular and ophthalmologic complications. While PXE is a systemic condition, this article will emphasize cardiovascular findings, especially those relevant to the presented case, such as peripheral vascular symptoms and gastrointestinal involvement.

## Case presentation

**Figure 1:**
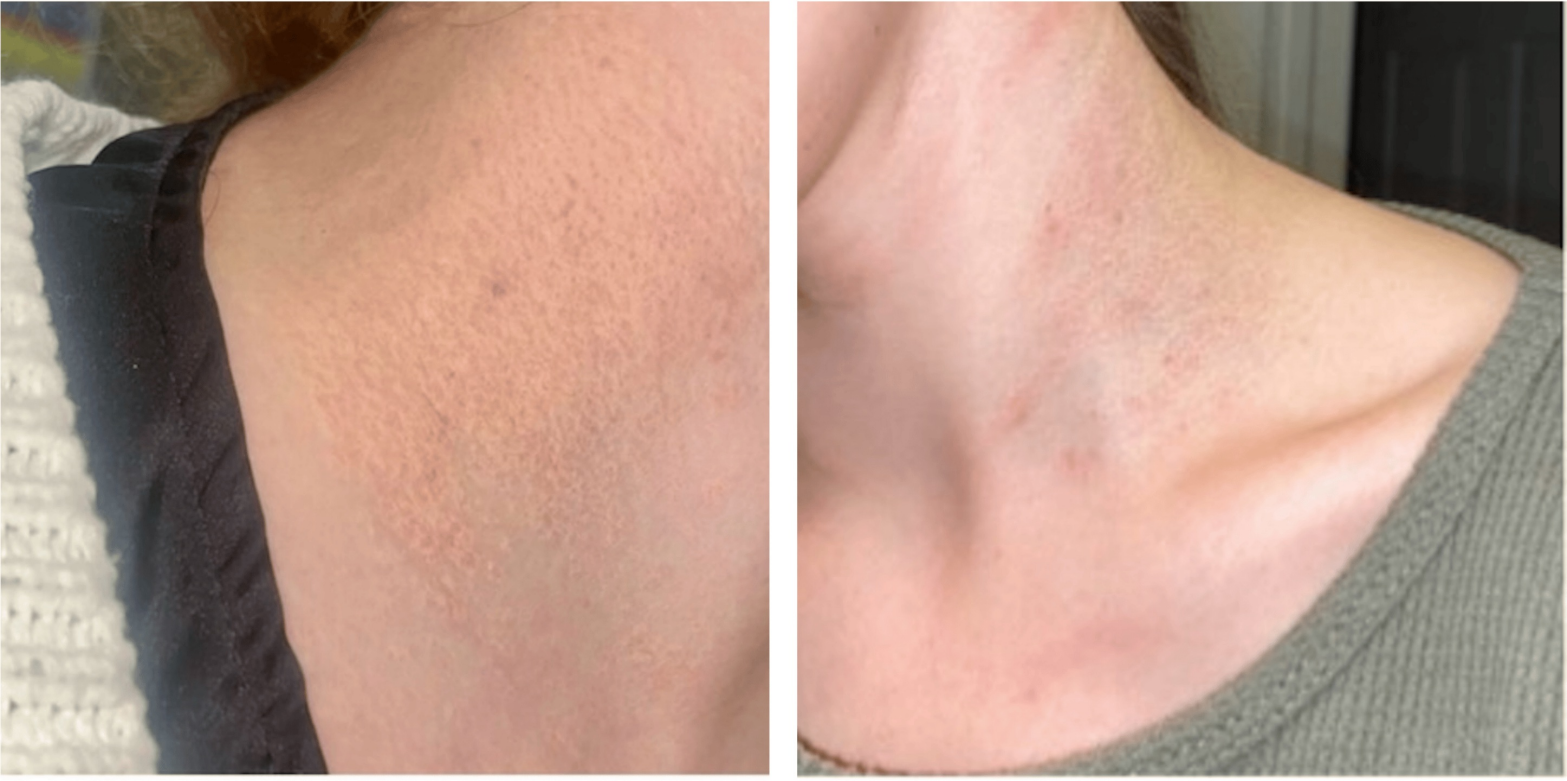
Cutaneous manifestations of pseudoxanthoma elasticum (PXE) Hyperpigmented, yellow-brown plaque along the left neck in a patient with PXE. The lesion demonstrates characteristic skin changes due to elastic fiber degeneration, commonly seen in PXE, which can also present with systemic vascular and ocular involvement.

A 22-year-old female patient with no significant past medical history presented to dermatology for a multi-year history of hyperpigmented, yellow-brown plaques on her neck, which later appeared on the axilla, elbows, and inguinal skin (Figure 1). A biopsy of her neck lesion demonstrated mild vascular hyperplasia of the dermis with a zone of irregular thickened and fragmented elastic fibers with haphazard arrangement; findings consistent with PXE. After the diagnosis of PXE was made, she was referred to ophthalmology. Despite the absence of visual symptoms, an ophthalmology referral was warranted given the frequency of ocular involvement in the disease [4]. Fundoscopic exam revealed classic findings of PXE: peau d’orange appearance and angioid streaks.

**Figure 2:**
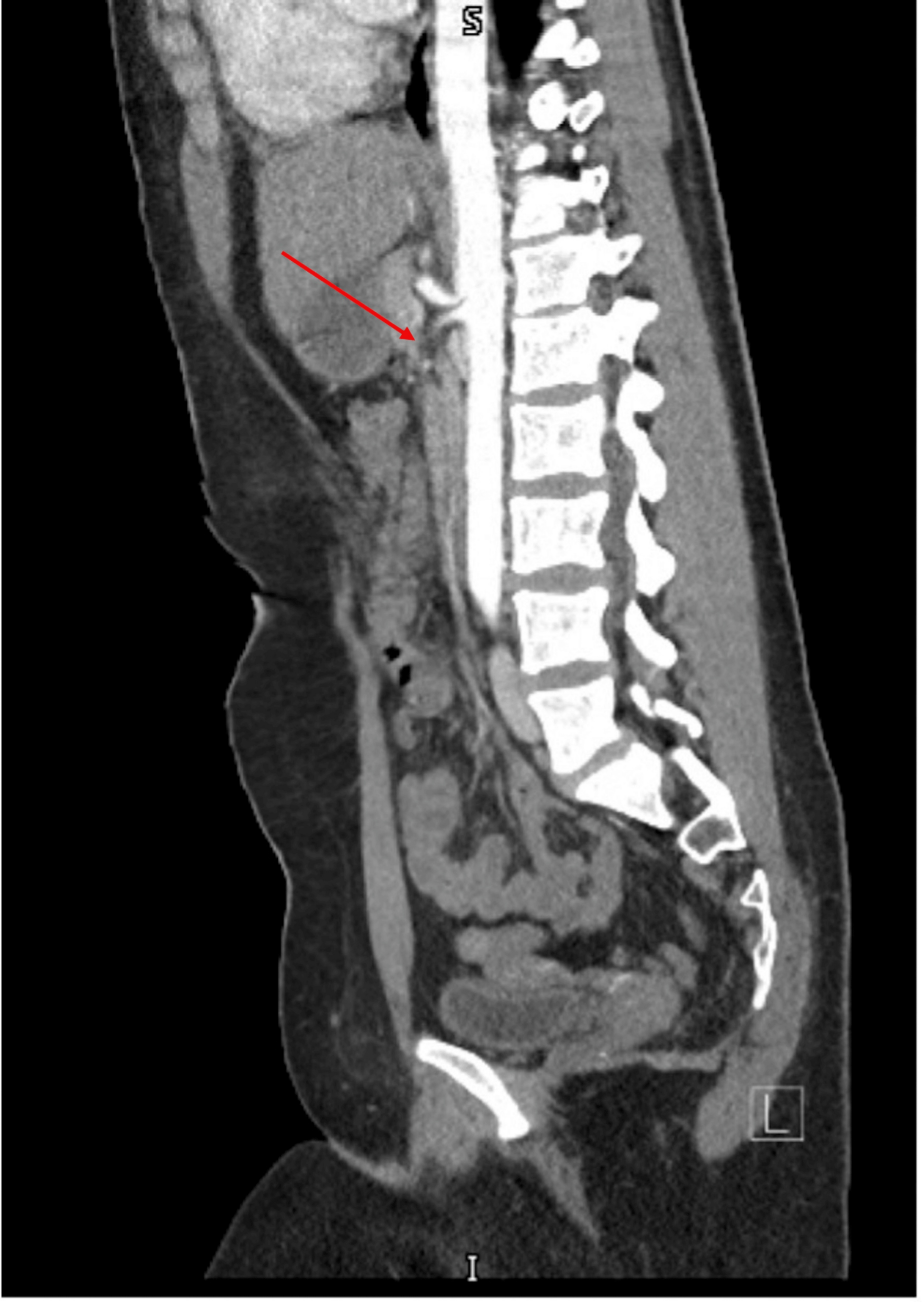
CT angiographic findings in median arcuate ligament syndrome (MALS) Sagittal view of a CT angiogram of the abdomen demonstrating the characteristic morphology of the celiac trunk in MALS. The superior compression of the celiac artery by the MAL results in narrowing and a hooked appearance of the vessel.

The patient also reported abdominal bloating and early satiety. A CT angiogram of the abdomen revealed a morphologic appearance of the celiac trunk consistent with median arcuate ligament syndrome (MALS) (Figure 2). Follow-up mesenteric Doppler ultrasound showed elevated velocities in both the celiac and superior mesenteric arteries at rest, supporting the diagnosis. These vascular findings, along with reported bilateral distal lower extremity cramping, prompted consultation with cardiology and vascular surgery. Although genetic testing for ABCC6 was not performed, the clinical diagnosis of PXE was made based on characteristic dermatologic, histopathologic, and ophthalmologic findings. The patient was advised to follow up annually with dermatology, cardiology, and ophthalmology.

The patient continues to follow up annually with ophthalmology, cardiology, dermatology, and gastroenterology for multidisciplinary surveillance. Ophthalmologic monitoring includes dilated fundus exams to assess for progression of angioid streaks or choroidal neovascularization. Cardiovascular follow-up focuses on vascular status and symptom monitoring for peripheral artery disease, while dermatologic surveillance tracks for new or evolving lesions. Her presenting plaques have remained stable. Gastroenterology follow-up has been conservative, as her symptoms of early satiety and abdominal bloating have improved without intervention. She reports intermittent leg cramping, which has not progressed and is currently managed with activity modification. She is not on pharmacologic treatment at this time and remains active in daily life and employment.

## Discussion

Our case demonstrates a systematic workup for a patient with newly diagnosed PXE, a condition that can result in significant complications involving the eye, cardiovascular system, and gastrointestinal tract. Prompt referral to specialists is necessary to reduce morbidity and mortality in PXE patients.

Genetic studies indicate that PXE is inherited in an autosomal recessive pattern. However, some studies have shown that heterozygous individuals may also exhibit phenotypic traits associated with PXE [4]. The most common genetic variants associated with PXE are located on the ABCC6 gene. This gene, located on 16p13.11, encodes a protein that is a member of the ATP-binding cassette (ABC) subfamily C (CFTR/MRP). The ABCC6 protein is primarily expressed in the liver, kidneys, blood vessels, and skin [5]. The role of the ABCC6 transporter is not fully understood; however, it is known to translocate various molecules across cell membranes. Defects in this transporter result in abnormal calcification and changes in elastic fibers within tissues. In this case, the patient reported no known family history of PXE or related symptoms in first-degree relatives. Her parents did not undergo genetic testing, and no clinical signs of PXE have been documented in family members to date.

Early skin lesion diagnosis can lead to appropriate dermatologic care to manage cosmetic and symptomatic concerns [6,7]. Regular ophthalmic exams are essential, regardless of ocular symptoms, due to potential vision-threatening complications such as choroidal neovascularization or the development of angioid streaks [8,9]. Cardiovascular evaluations are vital to decrease the progression of arterial calcification, hypertension, stroke, and myocardial infarction [9,10]. Multidisciplinary involvement can reduce the risk of major events by addressing modifiable risk factors such as blood pressure, lifestyle, and cholesterol [10].

Despite increasing recognition of PXE, its systemic complications, particularly cardiovascular, remain incompletely understood. Our team conducted an extensive review of the cardiovascular manifestations associated with PXE, providing a comprehensive overview of current understanding and management strategies. In our patient, baseline cardiovascular evaluation included normal vital signs, an unremarkable electrocardiogram (ECG), and routine laboratory studies, including complete blood count and metabolic panel within normal limits. No clinical or imaging evidence of coronary, valvular, or large vessel disease was identified at the time of evaluation.

Mendelsohn et al. (1987) performed autopsies on three PXE cases, revealing unique tunica intima modifications resembling hyaline arteriosclerosis [10]. They also identified fragmentation, degeneration, and calcification of the elastic laminae within the tunica media, features reminiscent of Monckeberg’s arteriosclerosis in medium-sized vessels, as well as significant atherosclerotic plaques in large vessels. Additionally, the endocardial elastic layer appeared disorganized, fragmented, and calcified. These histologic findings suggest a systemic metabolic defect that may predispose PXE patients to premature vascular aging, with increased risk for sudden cardiac death, restrictive cardiomyopathy, and heart failure with preserved ejection fraction.

Fibroelastic thickening of the endocardium in PXE can cause structural changes, disrupting electrical conduction and predisposing patients to arrhythmias. It also reduces ventricular compliance during diastole, leading to increased filling pressures and eventually clinical heart failure [11]. Campens et al. investigated cardiovascular phenotypes in 23 ABCC6 mutation carriers, 32 PXE patients, and 28 healthy controls [3]. They found that PXE patients had impaired left ventricular diastolic function, reduced aortic elasticity, and a high prevalence of peripheral artery disease. Heterozygous carriers exhibited milder diastolic dysfunction and signs of accelerated atherosclerosis, including increased carotid intima-media thickness and altered pulse wave velocity. Saban-Ruiz et al. reported lower arterial elasticity, higher cardiac output, and increased total vascular impedance in PXE patients compared to controls [12]. Pizarro et al. used cardiopulmonary exercise testing to evaluate functional capacity in PXE, revealing significantly reduced peak work rate, oxygen uptake, oxygen pulse, and minute ventilation, suggesting substantial cardiocirculatory impairment without ventilatory limitation [13]. These findings underscore the need for further research to define cardiovascular screening and management strategies in PXE.

Increased fibroelastosis of the cardiac valves has been reported in PXE, most commonly affecting the aortic and mitral valves, and can lead to symptoms such as dyspnea, angina, palpitations, fatigue, and new-onset murmurs. Documented complications include aortic stenosis, severe aortic regurgitation, and dilation of the aorta and atria, sometimes progressing to aneurysm formation [3]. Early recognition and management are critical due to the broad spectrum of cardiovascular involvement. A baseline transthoracic echocardiogram (TTE) should be performed at diagnosis, followed by repeat TTE every one to two years or sooner if new symptoms develop or prior abnormalities are identified [13-15]. Given the potential for restrictive physiology and endocardial involvement, cardiac MRI may offer additional diagnostic value, particularly in complex cases, although its specific role in PXE has not been fully defined. Referral to structural heart specialists is recommended when significant valvular or aortic pathology is detected to guide appropriate interventions [13-15].

Managing cardiovascular complications in PXE involves standard cardiac therapies tailored to the unique challenges of the disease. Antihypertensive medications, lipid-lowering agents, antiplatelet therapy, and, in select cases, anticoagulation are used based on individual risk profiles. Anticoagulation may be considered in PXE patients with atrial fibrillation or documented thromboembolic events. However, warfarin is generally avoided due to its role in promoting vascular calcification and increasing the risk of hemorrhage in calcified vessels [16]. While robust data in PXE is lacking, direct oral anticoagulants (DOACs) may be preferred when anticoagulation is indicated, though caution is warranted given the absence of disease-specific trials. Management of angina and heart failure follows guideline-directed medical therapy, with interventional or surgical approaches considered based on severity [16]. Lifestyle modification, routine surveillance for vascular calcification, multidisciplinary care, and genetic counseling are essential components of long-term management [16,17].

Genetic counseling is crucial due to the phenotypic variations in PXE. De Vilder et al. investigated rare genetic variants in 11 PXE patients, identifying 20 potential modifier genes associated with cardiovascular disease. Four key genes (CSF1R, NLRP1, SELE, and TRPV1) were expressed in patients with varying cardiovascular phenotypes [14,17]. Further genetic sequencing may help clinicians risk-stratify PXE patients with increased cardiovascular risk [14,17]. Establishing care with specialists early can decrease morbidity and mortality [14].

Utani et al. examined 14 PXE patients to explore the link between skin and mucous membrane lesion distribution and cardiovascular disease prevalence. Lesions were assessed across six sites: oral mucosa, neck, periumbilical region, cubital fossa, axillae, and inguinal regions, yielding a distribution score from 0 to six (one point per site). Patients with PXE-associated cardiovascular disease had a significantly higher mean distribution score (5.7) compared to those without cardiovascular disease (1.8) [18]. Notably, both higher distribution scores and the presence of oral mucosal lesions were associated with cardiovascular disease. Applying this scale to our patient resulted in a score of four (lesions on the neck, inguinal region, elbow, and axilla), indicating a moderate risk profile.

While the study did not outline a formalized management pathway for higher-risk patients, this scoring method may be useful in guiding more proactive cardiovascular surveillance. For patients with elevated scores, clinicians may consider earlier or more frequent screening for hypertension, hyperlipidemia, peripheral artery disease, and, when appropriate, advanced testing such as stress echocardiography or vascular imaging.

## Conclusions

This case highlights the systemic nature of PXE and underscores the importance of a comprehensive, multidisciplinary approach. While PXE is often recognized for its dermatologic and ocular findings, its cardiovascular manifestations require vigilant evaluation and ongoing surveillance. The co-occurrence of a vascular anomaly, such as median arcuate ligament syndrome, in this patient may be coincidental; however, it raises questions about potential overlap that warrant further investigation. Early identification and coordinated management remain essential to mitigating complications and optimizing long-term outcomes.
